# High-throughput measurement of the content and properties of nano-sized bioparticles with single-particle profiler

**DOI:** 10.1038/s41587-023-01825-5

**Published:** 2023-06-12

**Authors:** Taras Sych, Jan Schlegel, Hanna M. G. Barriga, Miina Ojansivu, Leo Hanke, Florian Weber, R. Beklem Bostancioglu, Kariem Ezzat, Herbert Stangl, Birgit Plochberger, Jurga Laurencikiene, Samir El Andaloussi, Daniel Fürth, Molly M. Stevens, Erdinc Sezgin

**Affiliations:** 1 Science for Life Laboratory, Department of Women’s and Children’s Health, Karolinska Institutet, Solna, Sweden; 2 Department of Medical Biochemistry and Biophysics, Karolinska Institutet, Stockholm, Sweden; 3 Division of Infectious Diseases, Department of Medicine Solna and Center for Molecular Medicine, Karolinska Institutet, Stockholm, Sweden; 4 Department Medical Engineering, University of Applied Sciences Upper Austria, Linz, Austria; 5 Department of Laboratory Medicine, Karolinska Institutet, Huddinge, Sweden; 6 Medical University of Vienna, Center for Pathobiochemistry and Genetics, Institute of Medical Chemistry, Vienna, Austria; 7 LBG Ludwig Boltzmann Institute for Traumatology, Nanoscopy, Vienna, Austria; 8 Lipid Laboratory, Department of Medicine Huddinge, Karolinska Institutet, Stockholm, Sweden; 9 Science for Life Laboratory, Department of Immunology, Genetics and Pathology, Uppsala University, Uppsala, Sweden; 10 Department of Materials, Department of Bioengineering and Institute of Biomedical Engineering, Imperial College London, London, UK

**Keywords:** Biophysical methods, Membrane biophysics, High-throughput screening, Nanoscience and technology

## Abstract

We introduce a method, single-particle profiler, that provides single-particle information on the content and biophysical properties of thousands of particles in the size range 5–200 nm. We use our single-particle profiler to measure the messenger RNA encapsulation efficiency of lipid nanoparticles, the viral binding efficiencies of different nanobodies, and the biophysical heterogeneity of liposomes, lipoproteins, exosomes and viruses.

## Abstract (web summary)

Lipid nanoparticles, viruses, exosomes and liposomes are characterized by analysis of fluorescence fluctuations.

## Main

Physiological nanometer-sized particles in the human body are of importance for health and disease. For example, lipoproteins (5–80 nm) transport lipids to maintain cellular metabolism^1^; extracellular vesicles (EVs, <200 nm) take part in immune responses, cell–cell communication and signaling^2^; and viruses with an average size of 100–200 nm cause a variety of diseases. Moreover, synthetic liposomes and lipid nanoparticles (LNPs) are widely used in drug delivery and vaccines^3^. Analysis of their content and biophysical properties can shed light on their structure, function and behavior in health and disease. Most of the existing methods to study bioparticles rely on biochemical analysis, mass spectroscopy, total internal reflection fluorescence microscopy and conventional flow cytometry. Biochemical methods and mass spectroscopy are bulk methods (that is, they lack single-particle sensitivity). Total internal reflection fluorescence microscopy provides an excellent signal-to-noise ratio in a truly single-particle manner; however, it requires fixation of the vesicles on the surface and yields low-throughput data. Flow cytometry, on the other hand, is a high-throughput method; however, it is generally suitable only for cell-sized objects. Recently, there have been attempts to analyze small EVs with flow cytometry^4–9^, and there is ongoing development of ‘nano-flow’ devices that rely on microfluidic equipment^10,11^. However, these methods are still limited to an average particle size of >200 nm and require dedicated and often costly equipment.

**Fig. 1:**
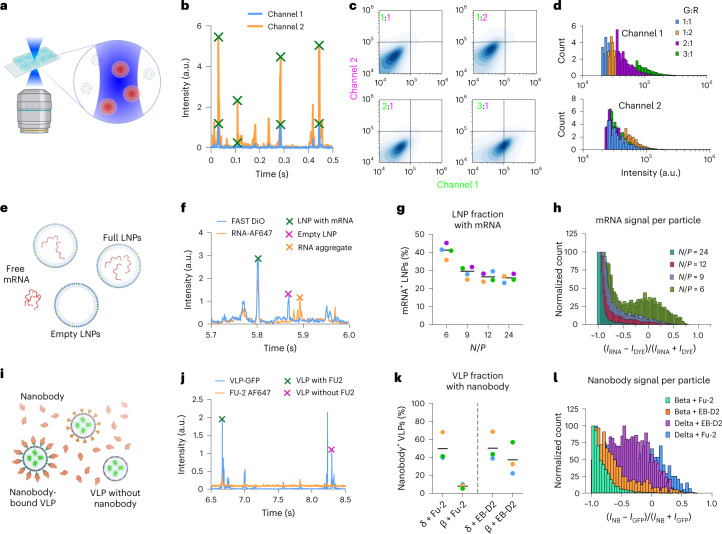
SPP for content analysis. **a**, SPP setup. **b**, Fluorescence intensity traces and peak calling. **c**, Two-dimensional density map of fluorescence intensities for liposomes loaded with FAST DiO and Abberior Star Red DPPE at different ratios. **d**, Intensity distributions for each channel for the samples shown in **c**. **e**, Scheme for mRNA encapsulation of LNPs. **f**, Representative peaks for possible scenarios. **g**, LNP fraction with mRNA signals for different *N*/*P* ratios (colors represent the mean of one replicate). **h**, Ratiometric histogram of mRNA versus lipid dye intensity for single LNPs. **i**, Scheme for nanobody binding to VLPs. **j**, Representative peaks for possible scenarios. **k**, VLP fraction with nanobody signal for different nanobodies and different variants (colors represent the mean of one replicate). **l**, Ratiometric histogram of nanobody versus lipid dye intensity for single VLPs. All histograms are representative of three replicates.

We have designed a method, single-particle profiling (SPP), based on analysis of fluorescence fluctuations of thousands of diffusing particles in solution, recorded with a commercially available confocal microscope (Fig. 1a). Briefly, fluorescently labeled particles diffuse through the diffraction-limited observation volume, where the fluorescence emissions from multiple channels are monitored continuously (as in fluorescence cross-correlation spectroscopy^12,13^). Unlike fluorescence cross-correlation spectroscopy, which reduces all fluctuations into a single curve^14^, we identify individual peaks in intensity fluctuations in multiple channels using a custom, freely available Python script (Fig. 1b; the software and video manual can be downloaded from GitHub). Based on the intensity of each individual peak in multiple channels, we construct a density plot (Fig. 1c) and histograms for each individual channel (Fig. 1d). Therefore, like flow cytometry, this approach can be used to measure the fluorescence intensities in single nanometer-sized particles smaller than 200 nm. Such information can be used for content measurement and biophysical profiling of particles.

First, to demonstrate the ability of SPP in content profiling, we prepared liposomes loaded with green molecules and/or red molecules incorporated into the lipid membrane. Single-loaded or double-loaded (only green dye, only red dye or both) confirmed the applicability of our methodology for content profiling (Supplementary Fig. 1). To test the content differences that were accessible to SPP, we changed the ratio of the green (FAST DiO) and red (Abberior Star Red DPPE) signals in liposomes (1:1, 1:2, 2:1 and 1:3) and found that these changes were successfully captured by SPP (Fig. 1c,d). To show the high-throughput capacity of SPP, we performed this experiment in multiwell plates (Supplementary Fig. 2).

In SPP measurements, each peak is analyzed with respect to its brightness, width and co-occurrence with another color (Supplementary Fig. 3). Peak brightness can be used to determine the clustering of particles (Supplementary Fig. 4) and, combined with peak width analysis, it can detect the presence of small numbers of large particles or aggregates (Supplementary Fig. 5). Furthermore, co-occurrence of the same peak in multiple channels and analysis of corresponding intensities provide information on particle content. Conventional codiffusion analysis fails to evaluate co-occurrence robustly in heterogenous samples because a single bright peak (which represents a large-sized impurity in the sample) skews the cross-correlation analysis (Supplementary Fig. 6). To use this advantage of SPP, we applied it to two biological challenges, measuring (1) messenger RNA (mRNA) encapsulation efficiency of LNPs and (2) antibody binding to virus particles.

LNPs are widely used as drug delivery and vaccine delivery agents^15^. One of the crucial parameters in the effectiveness of LNP-based treatments is the payload encapsulation efficiency. We used SPP to measure the encapsulation efficiency of LNPs loaded with mRNA (Fig. 1e). Current methods for measurement of mRNA encapsulation efficiency of LNPs are based on bulk measurements using RNA-binding dyes such as RiboGreen (Supplementary Fig. 7). This assay measures the fluorescence signal from the RiboGreen dye in the sample before versus after detergent treatment; the before and after signals are proportional to nonencapsulated mRNA in solution versus total mRNA, respectively. This ratio is termed the ‘encapsulation efficiency’. It is a measure of the percentage of mRNA encapsulated in LNPs at the population level; however, it does not yield insights into the fraction of empty versus full LNPs. As such, even if the RiboGreen assay shows 100% encapsulation efficiency (that is, all mRNA in the sample is inside LNPs), it is still possible that only a fraction of the LNPs carry all the cargo, whereas another pool is empty. Using SPP, however, we directly visualized and quantified encapsulation by measuring the mRNA and lipid dye signal co-occurrence for each particle (Fig. 1f). Control LNPs with two lipid dyes showed ≈100% co-occurrence, confirming the robustness of SPP for such measurements (Supplementary Fig. 7). In the encapsulation analysis, each LNP (blue peaks in Fig. 1f) was quantitatively analyzed with respect to its mRNA content (that is, whether it contained mRNA and how much). We calculated the fraction of LNPs showing an mRNA signal, that is, the full LNP fraction (Fig. 1g), as well as the encapsulation heterogeneity, that is, how much mRNA each LNP contained (Fig. 1h). Here, a value of −1 corresponds to empty LNPs, and higher values indicate more mRNA inside LNPs; see Methods for calculations. By changing the charge ratio of the ionizable lipid–mRNA (*N*/*P* ratio), we found that the most efficient encapsulation (in terms of both the fraction of full LNPs and the amount of mRNA inside each LNP) was achieved at *N*/*P* = 6. This shows that LNP formulation parameters affect both the cargo encapsulation and the amount loaded per LNP, which have implications for the performance of LNPs as drug carriers. Unlike currently prevailing bulk methods such as the RiboGreen assay, which can only give an overall percentage for the loading efficiency without any insight into the cargo distribution, SPP provides single-particle information for use in the development and quality control of LNP-based drug formulations.

Virus neutralization by antibodies is key for immunity. It is essential to swiftly determine whether certain antibodies bind the strain of interest. We applied SPP to evaluate the binding efficiency of nanobodies specifically developed to bind the SARS-CoV-2 Spike protein. To this end, we generated different variants of SARS-CoV-2 virus-like particles (VLPs, beta and delta) carrying GFP and incubated them with nanobodies Fu2 and EB-D2 (which target the receptor-binding domain and an epitope outside this domain, respectively) site-specifically conjugated to a fluorescent dye^16–18^ (Fig. 1i). SPP analysis showed virus peaks that did or did not co-occur with nanobody peaks (Fig. 1j). Quantification of the co-occurrence showed that ≈50% of delta and only ≈10% of beta VLPs were bound by Fu2. On the other hand, ≈50% of delta and ≈40% of beta VLPs were bound by the EB-D2 nanobody (Fig. 1k). Normalized intensity histograms were used to illustrate how much antibody bound to each type of virus particle; the largest amount of antibody was bound to delta–Fu2 particles, followed by delta–EB-D2, beta–EB-D2 and beta–Fu2, in that order (Fig. 1l). Thus, SPP is sensitive enough to detect differences in binding of nanobodies to different viral variants, paving the way for antibody neutralization screening.

**Fig. 2:**
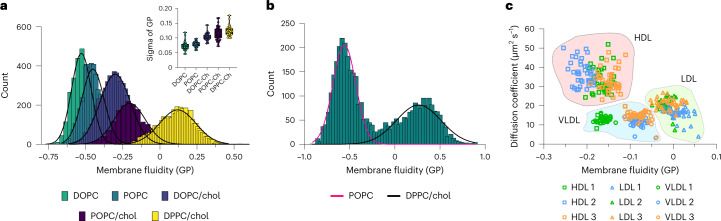
Biophysical properties of nano-sized bioparticles determined by SPP. **a**, Histograms of GP for liposomes of different lipid compositions; inset shows the *σ* of the GP distribution (*n* = 3 biological replicates, *n* = 40 technical replicates for all except *n* = 39 for DOPC and POPC/chol; box indicates 25–75%, whiskers indicate minimum and maximum values, lines mark the mean values). **b**, Representative GP histogram for the mixture of liposomes with distinct composition, showing two distinct populations. **c**, Dot plot of diffusion coefficient versus GP for lipoproteins from three different donors. All histograms are representative of three replicates.

Co-occurrence of particle fluorescence signals in multiple channels can also be used to study biophysical properties of nano-sized bioparticles using ratiometric environment-sensitive probes (Supplementary Fig. 8). To demonstrate the ability of SPP in such biophysical profiling, we prepared liposomes of distinct lipid compositions and supplemented them with 0.1 mol.% of the ratiometric dye Nile Red 12S (NR12S, Supplementary Fig. 1). The emission spectrum of this dye is sensitive to the fluidity of the lipid environment, which is measured with an index called generalized polarization (GP). The numerical value of GP ranges between +1 and −1 and is inversely proportional to membrane fluidity (for example, higher GP indicates lower fluidity). We prepared liposomes of different membrane fluidities by using saturated lipids, unsaturated lipids (18:1/18:1 1,2-dioleoyl-*sn*-glycero-3-phosphocholine (DOPC), 18:1/16:0 1-palmitoyl-2-oleoyl-*sn*-glycero-3-phosphocholine (POPC), 16:0/16:0 dipalmitoylphosphatidylcholine (DPPC)) and cholesterol in various combinations. As expected, membrane fluidity increased with increasing lipid saturation degree and cholesterol content (Fig. 2a and Supplementary Fig. 9). The single-particle capability of SPP allowed us to perform advanced statistical analyses of the GP data and histograms. For example, sigma (*σ*) of the GP histogram provides information on the heterogeneity of the sample, that is, a smaller *σ* means more homogeneity. This was demonstrated for the liposome mixtures (inset graphs in Fig. 2a and Supplementary Fig. 9). The biophysical heterogeneity of the liposomes increased with the complexity of the lipid mixture. A key advantage of SPP compared with bulk techniques is its ability to extract single-particle readings from thousands of particles, allowing us to dissect multicomponent mixtures. To show this, we prepared a mixture consisting of POPC liposomes and DPPC–cholesterol (DPPC/chol) liposomes. Analysis of this mixture indeed revealed the presence of two liposome populations: one population of low-order (POPC) liposomes and a second population of high-order (DPPC/chol) liposomes (Fig. 2b). Moreover, even when mixed populations are similar in their biophysical properties and cannot be easily separated using multicomponent analysis, one can still analyze the heterogeneity by measuring the *σ* of the GP distribution (Supplementary Fig. 10).

One of the advantages of SPP compared with flow cytometry tools is its ability to measure diffusion of particles as it is based on fluorescence fluctuations. Two parameters instead of one generally provide better separation power (Supplementary Fig. 11). Here, diffusion of particles (which is directly related to particle size; Supplementary Fig. 12) can be used as an additional parameter to distinguish different particles. To demonstrate this, we used blood plasma from healthy donors and isolated three major types of lipoproteins (LPs): high-density lipoprotein (HDL), low-density lipoprotein (LDL) and very low-density lipoprotein (VLDL). LP heterogeneity is a critical factor in cholesterol homeostasis and in several disorders including cardiovascular diseases^19^. We labeled all LPs with NR12S and performed SPP and multiparameter analysis. LDL and VLDL particles were not clearly distinguishable using either diffusion or GP (that is, there were notable overlap for both parameters), but combining these parameters allowed us to discriminate between the two (Fig. 2c and Supplementary Fig. 13). Moreover, donor-to-donor differences were evident from these analyses. We performed similar analyses for liposomes, EVs, virus-like particles and LNPs (Supplementary Figs. 14–18). The results showed that SPP can provide multiparametric information (for example, biophysical properties and diffusion of particles).

Here, we present and validate an analysis method for high-throughput SPP. We show that (1) content and biophysical properties of nano-sized bioparticles can be studied with SPP in a single-particle and high-throughput manner; (2) sample heterogeneity can be studied using statistical analysis; and (3) multiple parameters (such as diffusion and fluidity) can be obtained for clustering analysis. We use these features of SPP to study the mRNA encapsulation efficiency of LNPs, viral binding efficiency of nanobodies and biophysical heterogeneity of nano-sized bioparticles. This method is based on commercially available and highly accessible confocal systems and has wide applicability for resolving the content and organization of nano-sized physiological particles. SPP does not require dedicated equipment and is not limited to fixed spectral regions owing to the detector flexibility of confocal systems. SPP provides information on lipid and protein content, biophysical parameters and polydispersity of nano-sized bioparticles, which can shed light on the roles of these particles in health and disease.

## Methods

### Preparation of liposomes

DOPC, POPC, DPPC and cholesterol were from Avanti Polar Lipids. We used the following fluorescent lipids and lipid-like probes: Abberior Star Red DPPE (Abberior), TopFluor Cholesterol (Avanti), FAST DiO (ThermoFisher) and NR12S (provided by A. Klymchenko, University of Strasbourg). Five lipid mixtures in chloroform at 1 mg ml^−1^ were prepared: pure DOPC, pure POPC, DOPC/chol (70:30 ratio), POPC/chol (70:30) and DPPC/chol (70:30). In addition, lipid mixtures with different cholesterol percentages were prepared: POPC/chol (95:5), POPC/chol (90:10), POPC/chol (85:15), POPC/chol (80:20), POPC/chol (75:25), POPC/chol (70:30), POPC/chol (65:35), POPC/chol (60:40), POPC/chol (55:45) and POPC/chol (50:50). Finally, mixtures containing POPC with different ratios of fluorescent lipids were prepared: POPC/FAST DiO/Abberior Star Red PE (99.8:0.1:0.1), POPC/FAST DiO/Abberior Star Red PE (99.7:0.1:0.2), POPC/FAST DiO/Abberior Star Red PE (99.7:0.2:0.1), POPC/FAST DiO/Abberior Star Red PE (99.6:0.3:0.1), POPC/TopFluor Cholesterol/Abberior Star Red PE (99.8:0.1:0.1), POPC/TopFluor Cholesterol (99.9:0.1) and POPC/ Abberior Star Red PE (99.9:0.1). Mixtures were dried under nitrogen flow, rehydrated with buffer (150 mM NaCl, 10 mM HEPES, 2 mM CaCl_2_) and vortexed harshly to form multilamellar vesicles. Then, the suspension of MLVs was sonicated at power 3, duty cycle 40%, for 10 min using a Branson Sonifier 250. The size of the resulting vesicles was checked using dynamic light scattering (Malvern Zetasizer). Liposomes were stored at 4 °C under nitrogen, and the solutions of liposomes (1 mg ml^−1^) were incubated with NR12S (1 µM in dimethyl sulfoxide (DMSO)) directly before profiling.

### Isolation of lipoproteins

Blood plasma was obtained from the blood transfusion station of Karolinska Hospital, Stockholm, donated by apparently healthy normolipidemic volunteers. Lipoprotein particles were isolated as previously described via sequential flotation ultracentrifugation^20^. Briefly, the density of blood plasma was sequentially adjusted using KBr to 1.019 g l^−1^, 1.063 g l^−1^ and 1.22 g l^−1^ in order to isolate VLDL, LDL and HDL, respectively. Lipoproteins were stored at 4 °C under nitrogen, and the solutions of LPs (1 mg ml^−1^ in phosphate-buffered saline) were incubated with NR12S (1 µM in DMSO) directly before profiling.

### Preparation of fluorescent RNA

Fluorescently labeled RNA was synthesized by in vitro transcription (E2040S, NEB Inc.). One microgram of a double-stranded DNA template containing the T7 RNA promotor upstream of the Firefly luciferase gene (FLuc, 1.8 kb length, N0426, NEB Inc.) was added together with NTPs, 0.75× T7 RNA polymerase buffer and 75 units T7 RNA polymerase in a 20 µl reaction volume. The reaction was incubated overnight at 37 °C. NTPs were at a final concentration of 7.5 mM except for UTP, which had a final concentration of 5 mM and was supplemented with 1.24 mM 5-ethynyl-UTP (CLK-T08, Jena Biosciences Gmb). The addition of 5-ethynyl-UTP resulted in alkyne-functionalized RNA, which was subsequently processed via Cu(I)-catalyzed (azide–alkyne) cycloaddition (CuAAC) to add fluorophores onto the RNA. After in vitro transcription, RNA was purified using a silica spin column (R1055, Zymo Research Inc.). Fluorophores (AZDye 647 and AZDye 488) were added to the RNA by CuAAC using fluorophores conjugated to azides with a copper-chelating system in their structure (1475 and 1482, Click Chemistry Tools Inc.). Ligand BTTAA (50 mM, CLK-067, Jena Biosciences) was allowed to react with 10 mM of CuSO_4_ (v800132, Sigma Aldrich) in nuclease-free water. The functionalized RNA was eluted in 22.2 µl of nuclease-free water and supplemented with 1.8 µl of 10 mM azide-fluorophore dissolved in DMSO (276855, Sigma Aldrich). Then, 60 mg of sodium l-ascorbate was dissolved in 1 ml of nuclease-free water. Immediately after the ascorbate had been fully dissolved, it was added to the BTTAA/CuSO_4_ mixture at a final concentration of 100 mM. Six microliters of the BTTAA/CuSO_4_/ascorbate reaction were then added to the azide-fluorophore and RNA reaction, which was degassed using argon, sealed with parafilm and incubated overnight. Labeled RNA was purified using a silica spin column (R1055, Zymo Research Inc.) and measured on a NanoDrop (ThermoFisher Inc.). The fluorescently labeled RNA was ethanol precipitated overnight using 3 M sodium acetate to create a pellet for further experiments.

For preparation of LNPs, 1-oleoyl-rac-glycerol (monoolein) was purchased from Sigma Aldrich (Merck), and cholesterol (ovine wool), 1,2-dioleoyl-3-trimethylammonium-propane (18:TAP; DOTAP) and 1,2-dimyristoyl-sn-glycero-3-phosphoethanolamine-*N*-[methoxy(polyethylene glycol)-2000] (14:0 PEG2000 PE; DMPE-PEG2000) were purchased from Avanti Polar Lipids. All components were used without further purification. To make lipid films, the lipids were dissolved in chloroform (10–20 mg ml^−1^) and mixed to the appropriate volumes to obtain the desired molar ratios (monoolein/cholesterol/DOTAP/DMPE-PEG2000: 60:30:10:2.5 mol.%). Lipid films were further supplemented with 0.5 mol.% FAST DiO dye. Chloroform was evaporated at room temperature overnight. Lipid films were subsequently stored under nitrogen and sealed at −20 °C until LNP preparation. Lipid films were defrosted and dissolved in absolute ethanol at a concentration of 16.7 mM. Cargo was dissolved in 0.025 M pH 4 sodium acetate buffer in concentrations giving *N*/*P* ratios of 0, 6, 9, 12 and 24 in the final LNP formulations. LNPs were formulated using a syringe pump (Pump 33 dual drive system, Harvard Apparatus). Both solutions were loaded in 2.5 ml Hamilton glass syringes. The total flow rate was 0.5 ml min^−1^, with LNP/cargo solutions mixed at a ratio of 1:3. We used a passive herringbone mixer chip (Darwin Microfluidics), and the sample collection was started after the first 15 s. Following the microfluidic formulation, the LNP samples were dialyzed overnight at room temperature against DPBS++ (Gibco, Thermo Fisher Scientific) in Slide-A-lyzer mini dialysis tubes (Thermo Fisher Scientific; 3.5 kDa cut-off) to remove the ethanol and reach neutral pH. The pH was checked with pH paper.

Dynamic light scattering was performed immediately after the dialysis using a Malvern Zetasizer Nano series (Nano ZS) instrument. LNPs (1 μl) were diluted in 499 μl DPBS++ (Gibco) and measured at 25 °C in low-volume cuvettes. Cargo loading was evaluated with a Quant-it RiboGreen RNA assay kit (R11490, Invitrogen) using the manufacturer’s protocol. For RiboGreen, the LNP samples were diluted 1:20 with the TE buffer from the kit, and measurements were conducted for both intact and lysed LNPs (lysis with 2% (v/v) Triton X-100 in TE). Cargo loading efficiency as a percentage was calculated using the formula (*c*_after lysis_ – *c*_before lysis_)/*c*_after lysis_ × 100% (*c* is cargo concentration). LNPs were stored at room temperature until the microscopy experiments were performed.

### Preparation of exosomes

EVs were prepared from HEK293-FS suspension cells (ThermoFisher, R79007), cbMSC (immortalized human cord blood-derived mesenchymal stromal cells, ATCC PCS-500-010), BJ-5ta (immortalized human fibroblasts ATCC CRL-4001) and THP-1 (human monocytic cells, ATCC TIB-202). Cell lines were cultured in the following media: cbMSCs were cultured in MEM-α modification medium (containing l-glutamine; ThermoFisher Scientific) supplemented with 5 ng ml^−1^ bFGF (Sigma, F0291). BJ-5ta fibroblast cells were cultured with a 4:1 mixture of Dulbecco’s medium (containing 4 mM l-glutamine, 4.5 g l^−1^ glucose and 1.5 g l^−1^ sodium bicarbonate) and Medium 199 (0.01 mg ml^−1^ hygromycin B/10687010, ThermoFisher); HEK293-FS cells were cultured in FreeStyle 293 Expression Medium (ThermoFisher Scientific) in 125-ml polycarbonate Erlenmeyer flasks (Corning) in a shaking incubator (Infors HT Minitron) according to the manufacturer’s instructions; and THP-1 cells were cultured in RPMI-1640 medium (containing Glutamax-I and 25 mM HEPES, Invitrogen). Unless otherwise indicated, all cells were supplemented with 10% fetal bovine serum (Invitrogen) and 1× antibiotic-antimycotic (ThermoFisher Scientific). All cell lines were grown at 37 °C, 5% CO_2_ in a humidified atmosphere and regularly tested for the presence of mycoplasma. For EV harvesting, cell-culture-derived conditioned medium was changed for OptiMem (Invitrogen) 48 h before harvest of conditioned media as described before^21^. Unless indicated otherwise, all conditioned media samples were directly subjected to a low-speed centrifugation step at 500*g* for 5 min followed by a 2,000*g* spin for 10 min to remove larger particles and cell debris. Precleared cell-culture supernatant was subsequently filtered through 0.22-µm bottle-top vacuum filters (Corning, cellulose acetate, low protein binding) to remove any larger particles. EVs were isolated by tangential flow filtration (TFF). For TFF EV isolation, precleared conditioned medium was concentrated via TFF using a KR2i TFF system (SpectrumLabs) equipped with modified polyethersulfone hollow fiber filters with 300 kDa membrane pore size (MidiKros, 370 cm^2^ surface area, SpectrumLabs) at a flow rate of 100 ml min^−1^ (transmembrane pressure 3.0 psi and shear rate 3,700 s^−1^) as described previously^21^. Amicon Ultra-0.5 10-kDa MWCO spin filters (Millipore) were used to concentrate the sample to a final volume of 100 µl. The sample was then loaded on a qEV column (Izon Science) and the EV fractions were collected according to the manufacturer’s instructions.

### Preparation of virus-like particles

HEK293T (ATCC CRL-3216) cells were cultured in Dulbecco’s modified Eagle medium supplemented with 10% fetal calf serum and confirmed to be mycoplasma-free. To produce pseudotyped nonfluorescent VLPs, cells were seeded at a confluency of ~70% in T75 flasks and cotransfected 6 h later with 8.2 µg of the lentiviral packaging vector psPAX2 (gift from Didier Trono (Addgene plasmid number 12260) and 10 µg of the respective viral surface protein using jetOptimus (Polyplus) according to the suppliers’ recommendations. Human codon usage optimized plasmids encoding SARS-CoV-2 spike variants delta and beta and Ebola GP were kindly provided by B. Murrell and J. Bodem, respectively. After 12 h, media were exchanged and VLPs were harvested twice after 24 h each time. Enrichment of VLPs was performed using Lenti-X Concentrator (Takara) according to the protocol provided by the manufacturer. Alternatively, to increase the purity, supernatants with VLPs were filtered through 0.45-µm polyethersulfone filters and subjected to ultracentrifugation using a 40% sucrose cushion.

To produce fluorescent VLPs, Hek293T cells were cotransfected using Lipofectamine 3000 and 15 µg of the delta-spike expression plasmid, 7.5 µg DNA encoding HIV Vpr-GFP (NIH HIV Reagent Program, Division of AIDS, NIAID, NIH: pEGFP-Vpr, ARP-11386, contributed by W. C. Greene) and 7.5 µg encoding a lentiviral packaging plasmid (psPAX2 was a gift from D. Trono; Addgene plasmid number 12260). Media were exchanged after 12 h. VLPs were harvested after 24 and 48 h and enriched 50-fold using Lenti-X Concentrator according to the protocol provided by the manufacturer (Takara). To generate VLPs with different spike-cleavage patterns, cells were kept in the presence of 50 µM furin inhibitor (Decanoyl-RVKR-CMK; Tocris: 3501) after the medium change.

### SPP measurements and analysis

SPP was performed using the setup for fluorescence correlation spectroscopy on a Zeiss LSM 780 microscope. A 488-nm argon ion laser was used for TopFluor Cholesterol, FAST DiO, GFP and NR12S, and a 633 nm He−Ne laser was used for ASR PE. A 40 × 1.2 NA water immersion objective was used to focus the light. The laser power was set to 0.1−0.5% of the total laser power, corresponding to 2−10 μW. The emission detection windows were set to 490–560 for TopFluor Cholesterol and 650–700 for ASR PE. Emission from NR12S was recorded simultaneously in both channels. Forty intensity fluctuation traces 15 s long were acquired for each sample.

Traces and curves were analyzed with a homemade Python program using the following Python packages: tkinter (v.8.6.10), matplotlib (v.3.3.4), lmfit (v.1.0.2), ttkwidgets (v.0.10.0), scipy (v.1.6.2), seaborn (v.0.11.1) and pandas (v.1.2.4). The source code and the standalone distributions for Windows and Mac are available at Github: https://github.com/taras-sych/Single-particle-profiler.

Briefly, individual peaks from the traces were identified, and intensities for these individual peaks were extracted with further calculation of GP if applicable. Furthermore, based on these values, dot plots or density plots were constructed. The description and guide to the program is available as a video tutorial. The link to the video is at https://github.com/taras-sych/Single-particle-profiler.

Moreover, diffusion analysis was performed using the same homemade software. Curves were fitted with the following three-dimensional diffusion:

$$G\left(\tau \right)=\frac{1}{N}{\left(1+\frac{\tau }{{\tau }_{D}}\right)}^{-1}{\left(1+\frac{\tau }{{{{AR}}^{2}\tau }_{D}}\right)}^{-\frac{1}{2}}$$

where *N* represents the number of fluorescent species within the beam’s focal volume. Next, the diffusion coefficients were calculated as follows:

$$D=\frac{{\omega }^{2}}{8\,\mathrm{ln}(2){\tau }_{D}}$$

where *w* corresponds to the full-width at half-maximum of the point spread function, ${\tau }_{D}$ is the diffusion time and *D* is the diffusion coefficient.

### Statistical analysis

For every box plot, the exact sample size is shown in brackets. The replicates are from repeated measurements of the same biological sample. The significance was determined in all cases by nonparametric one-way analysis of variance. For all box plots, the central line indicates the mean, the box indicates 25–75% and whiskers indicate minimum and maximum values of the data. Not significant, *P* > 0.05; *P < 0.05; **P < 0.01; ***P < 0.001; ****P < 0.0001.

### Reporting summary

Further information on research design is available in the Nature Portfolio Reporting Summary linked to this article.

## Online content

Any methods, additional references, Nature Portfolio reporting summaries, source data, extended data, supplementary information, acknowledgements, peer review information; details of author contributions and competing interests; and statements of data and code availability are available at 10.1038/s41587-023-01825-5.

### Supplementary information

Supplementary Information Supplementary Figs. 1–19.

Reporting Summary

## Data Availability

All raw data are available at FigShare 10.17044/scilifelab.20338869.

## Code availability

The source code of the script as well as the standalone distributions are available at GitHub: https://github.com/taras-sych/Single-particle-profiler.
