# Supplementary material for: High-throughput measurement of the content and properties of nano-sized bioparticles with single-particle profiler
Source: Nat Biotechnol. 2023 Jun 12;42(4):587–90. doi: 10.1038/s41587-023-01825-5 (PMC11021190; doi:10.1038/s41587-023-01825-5)
Supplement: Supplementary file 1 — Supplementary Figs. 1–19. [file 41587_2023_1825_MOESM1_ESM.pdf]

nature biotechnology

Brief Communication

https://doi.org/10.1038/s41587-023-01825-5

High-throughput measurement of the content and properties of nano-sized bioparticles with single-particle profiler

In the format provided by the authors and unedited

Supplementary Figures

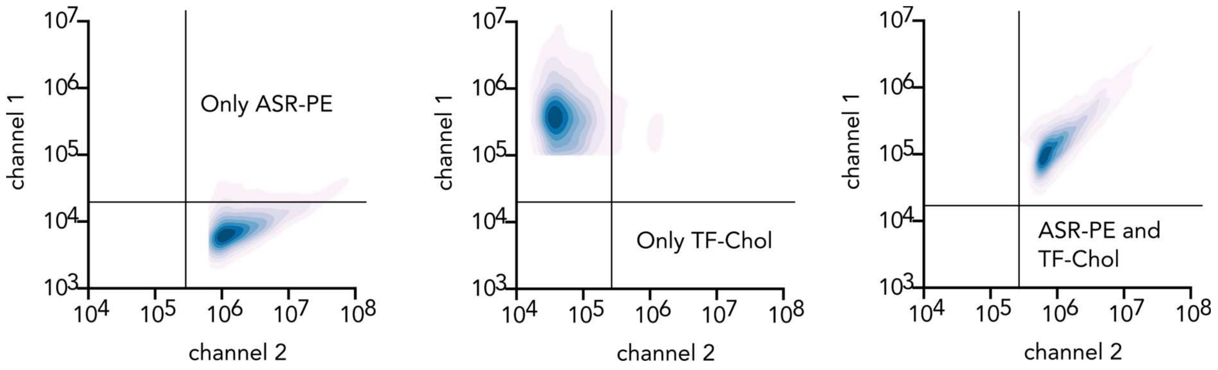

Supplementary Figure 1. Liposomes labelled with single fluorophore (TF-Chol: Topfluor Cholesterol; ASR-PE: Abberior STAR RED labelled phospholipid).

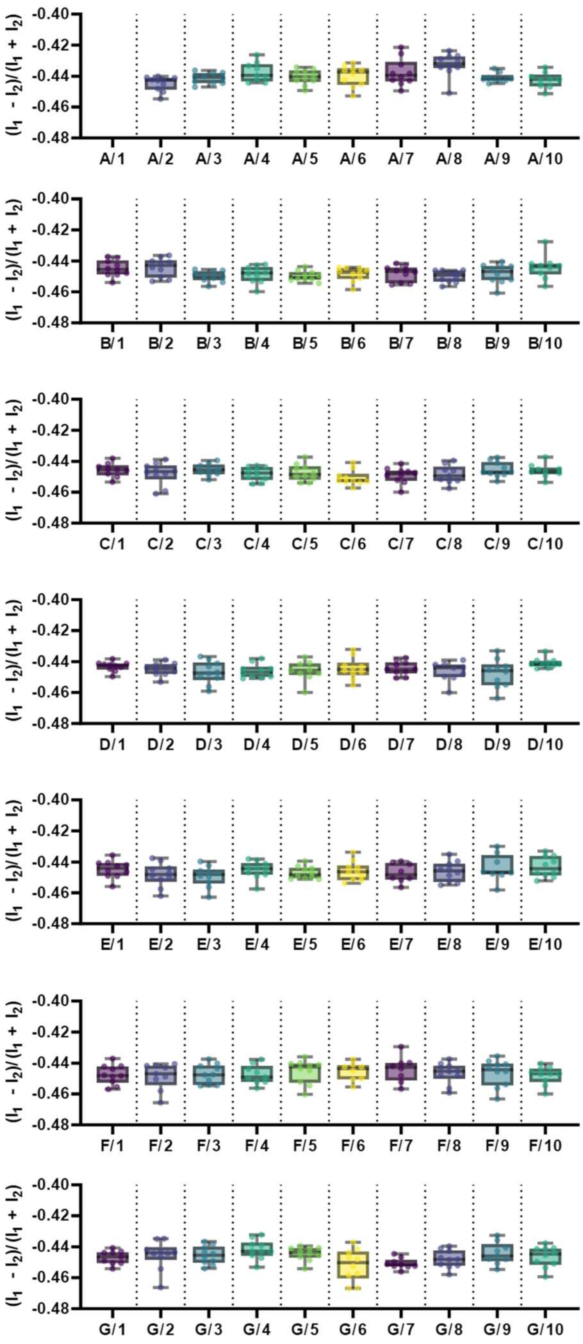

Supplementary Figure 2. High throughput analysis of multi-well plates with automated stage movement(n=3 biological replicate, numbers of technical replicates: 9 for A4, A9, A10, C1, C3, C8, C10, D5, D8, F2, F4, G1, G7, G9, 8 for E7, E9, F7, 10 for the rest box = 25-75%, whiskers = min-max, line marks mean value). The microscope stage moves once the chamber type and dimensions are introduced to the imaging program. Two channel recordings were done for 96-well plate with glass bottom and corrected intensity ratios were measured. Corner wells could not be reached due to physical hindrances by the stage and the objective.

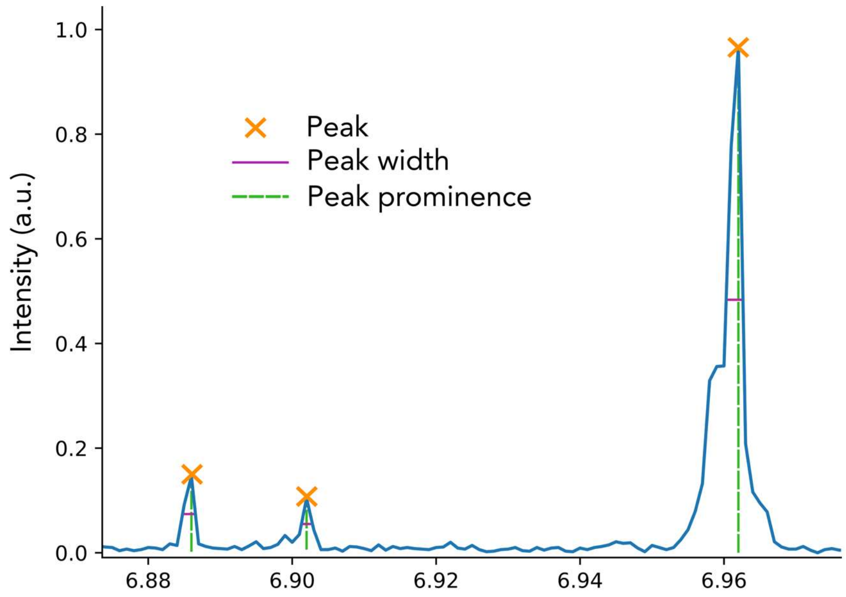

Supplementary Figure 3. Parameters that can be obtained for each peak.

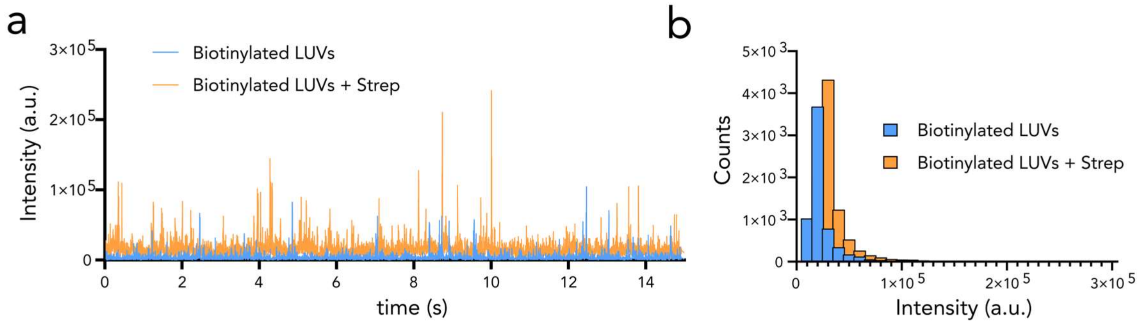

Supplementary Figure 4. Clusters of liposomes can be separated by brightness. a) Peaks caused by liposomes incorporated with fluorescent lipids and biotinylated lipids with and without streptavidin. Streptavidin causes aggregation, and the peak intensity increases accordingly; b) Intensity histograms before (blue) and after (prange) the introduction of streptavidin.

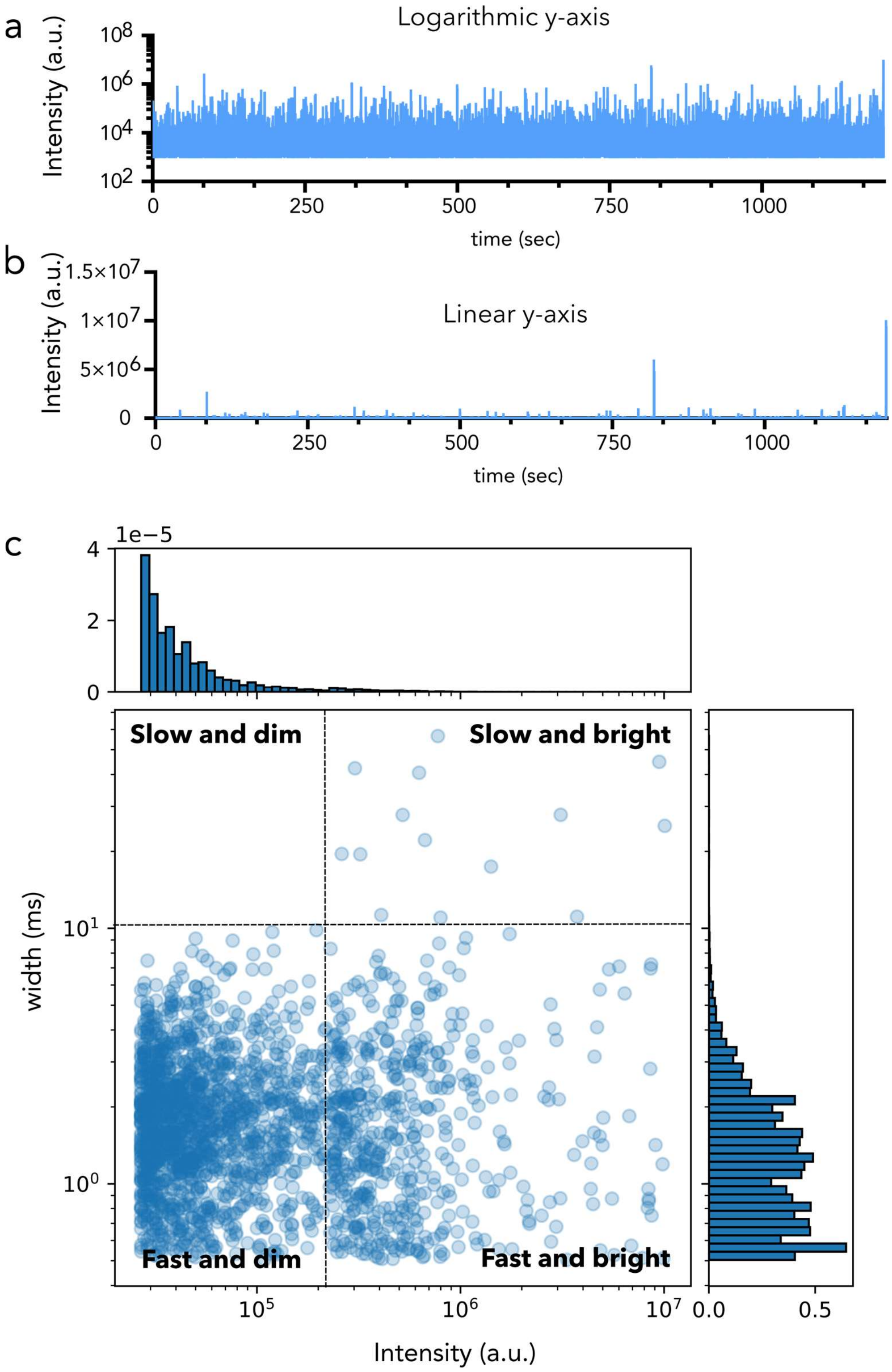

Supplementary Figure 5. Aggregated or big molecules can be distinguished clearly when peak width is combined with peak intensity. a, b) Peaks caused by an heterogenous liposome mixture (big and small liposomes) in linear vs logarithmic scales, respectively. c) Peak width vs intensity scatter plot shows slow/ bright particles compared to fast/bright and fast/dim particles.

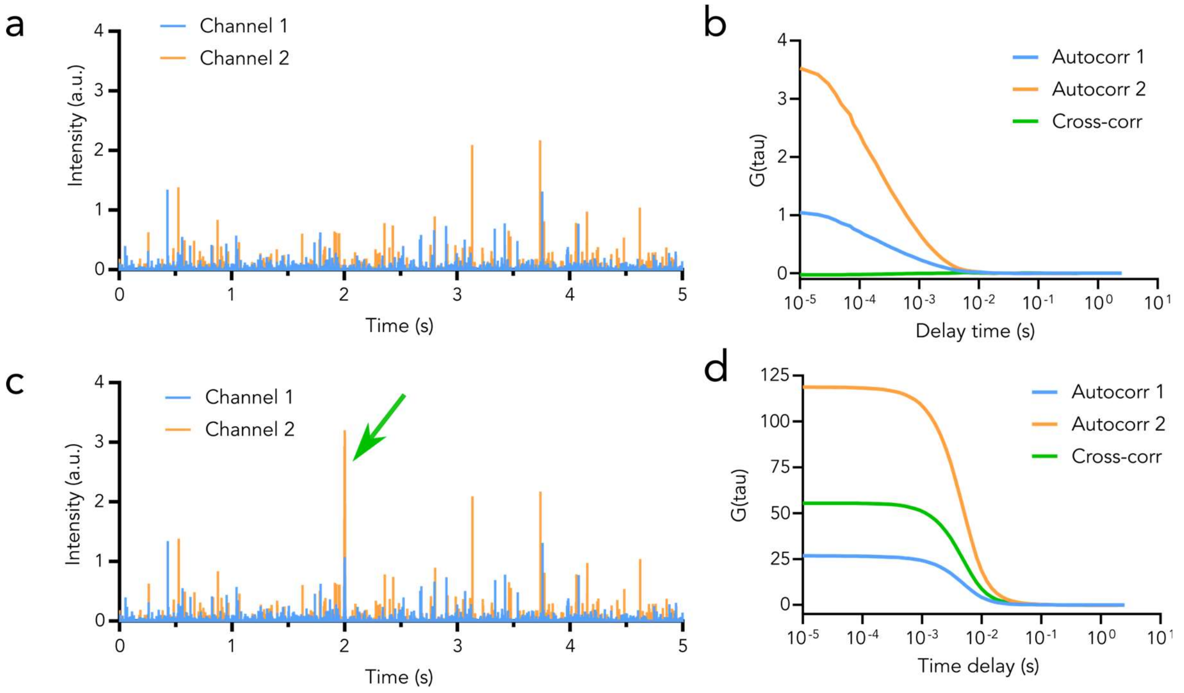

Supplementary Figure 6. Two colour fluorescence cross correlation spectroscopy cannot provide reliable information on number of co-occurrences in heterogeneous samples. a) Peaks caused by an homogenously distributed sample and b) cross-correlation curves showing no co-occurrences. c) the same sample with a single bright broad peak introduced artificially into both intensity traces at the same timepoint;d) the cross-correlation is now heavily biased by this single peak and shows 100% cross correlation. Data was generated by simulation via random-walk diffusion using home-made script in python. Script is freely available here: https://github.com/taras-sych/FCCS_simulation

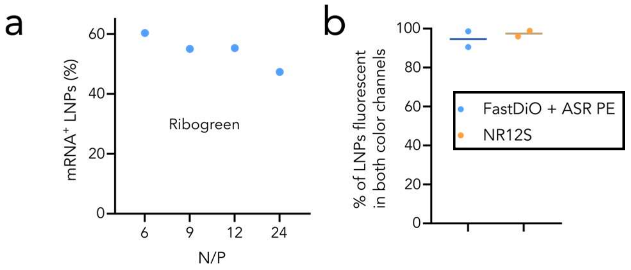

Supplementary Figure 7. LNP control experiments. a) RiboGreen analysis of LNPs with different N/P ratio. b) LNPs with green (Fast-DiO) and red lipid dyes (Abberior StarRed-PE) or a single dye with two emissions (NR12S) as a positive control for co-occurrence which show 100% co-occurrence.

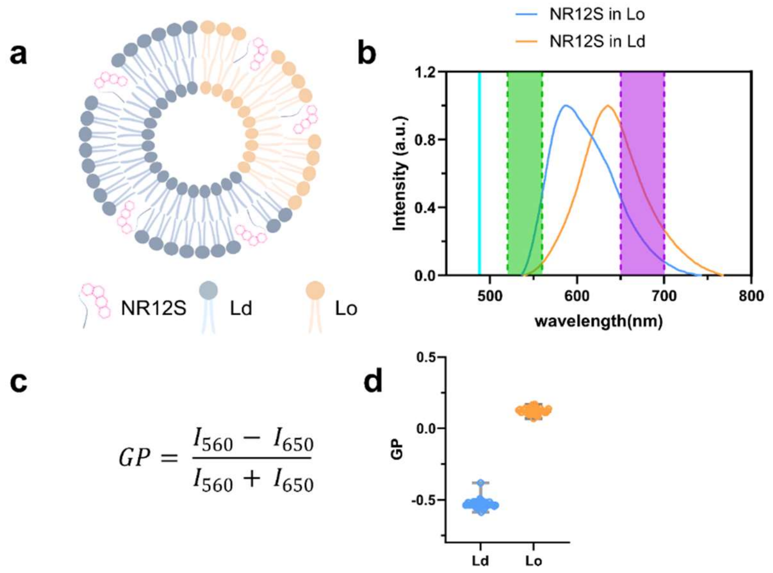

Supplementary Figure 8. Measurement of membrane order using environmental sensitive dye. a. Lipid bilayer consisting of two different domains, liquid ordered (Lo) and liquid disordered (Ld) labeled with NR12S; **b.** Fluorescence spectra of NR12S in Lo and Ld. Cyan line shows the excitation wavelength as well as green and magenta bands display the widths of the detection channels used for quantification in c; **c.** Quantification of the generalized polarization of NR12S spectra shown in b; **d.** GP of Lo and Ld (n=3 biological replicate, n=40 technical replicates, box = 25-75%, whiskers = min-max, line marks mean value).

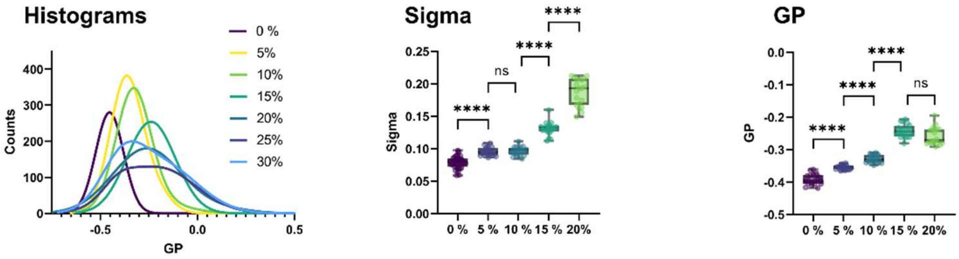

Supplementary Figure 9. LUVs with different content of cholesterol. Studies of mixtures of pure POPC with POPC/chol LUVs of different cholesterol content: GP histogram of mixtures, sigma of distribution, mean gp for single component distributions (n=3 biological replicate, n=20 technical replicates, box = 25-75%, whiskers = min-max, line marks mean value), statistical significance was probed by Kruskal-Wallis nonparametric test with multiple comparisons, **** corresponds to p<0.0001, ns for p>0.05

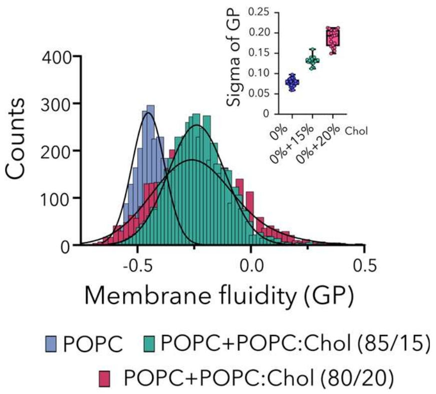

Supplementary Figure 10. LUVs with different content of cholesterol. Data for binary mixture of pure POPC liposomes and POPC/chol liposomes with different percentage of cholesterol. GP histograms show one population for 0% chol – 20% chol. Even where multiple populations cannot be resolved at low cholesterol mixtures, heterogeneity manifests in broadening of the distribution as reflected by the sigma values (n=3 biological replicate, n=20 technical replicates, box = 25-75%, whiskers = min-max, line marks mean value).

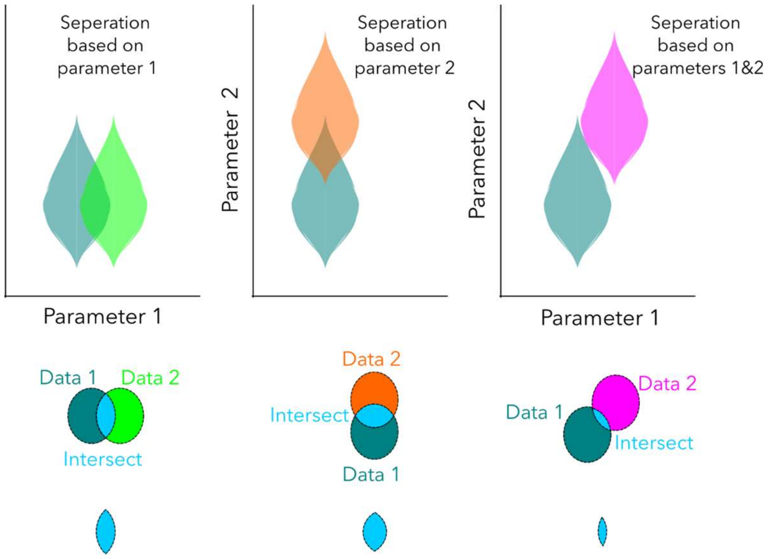

Supplementary Figure 11. Two parameters instead of one has higher separation power, with less overlap.

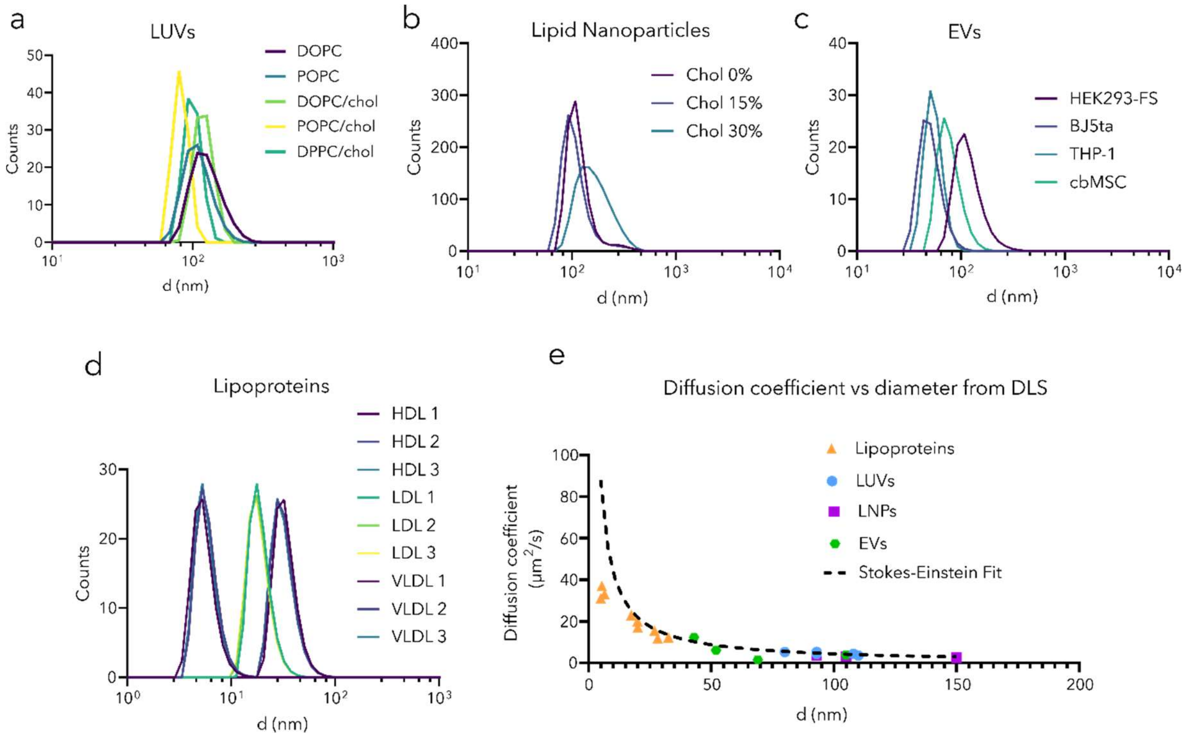

Supplementary Figure 12. Diffusion vs size from SPP measurements. a-d) DLS size measurements for liposomes, LNPs, EVs and lipoproteins. e) The relationship between the DLS size measurements and diffusion coefficient we obtained from SPP. Estimation is robust for all particles except HDLs.

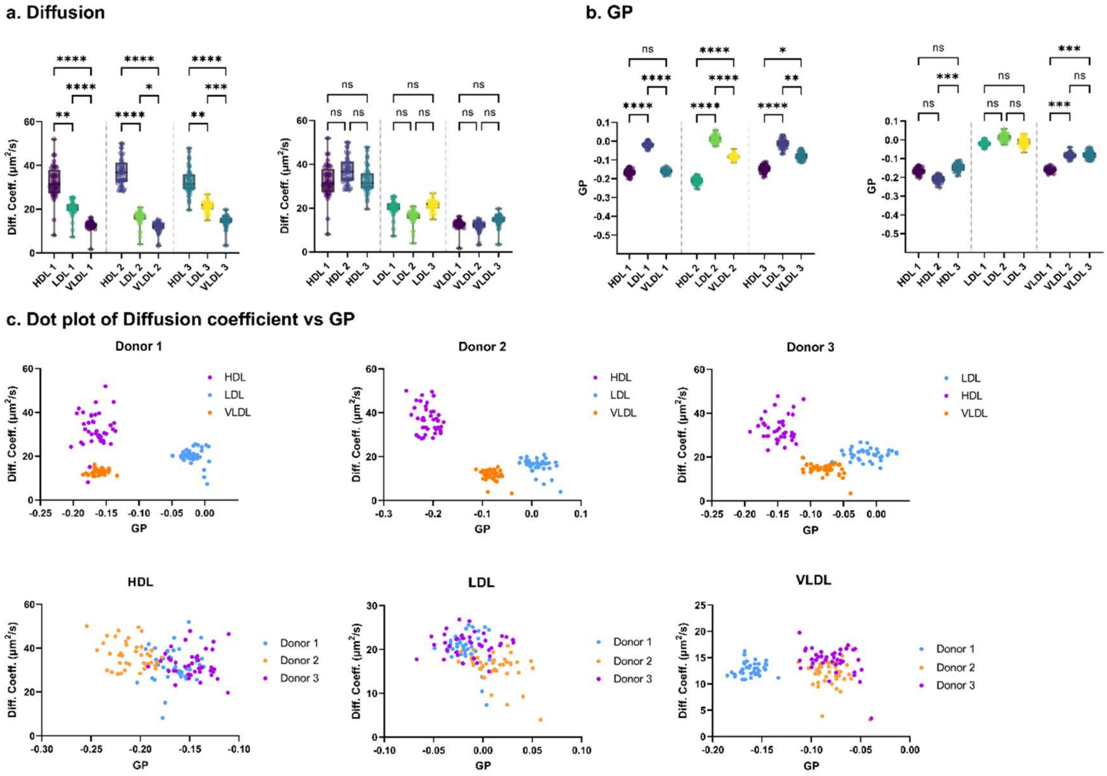

Supplementary Figure 13. Lipoproteins from healthy individuals. LPs were isolated from blood plasma of healthy individuals; **a.** Diffusion coefficient of lipoproteins; Left and right panel contain the same information, but on left panel it is sorted by donor and on right panel – by type of lipoprotein. **b.** GP of lipoproteins; Left and right panel contain the same information, but on left panel it is sorted by donor and on right panel – by type of lipoprotein (n=3 biological replicate, n=40 technical replicates, box = 25-75%, whiskers = min-max, line marks mean value), statistical significance was probed by Kruskal-Wallis nonparametric test with multiple comparisons, **** corresponds to p<0.0001, *** corresponds to p<0.001, ** corresponds to p<0.01, * corresponds to p<0.05 ns to p>0.05. **c.** Dot plot of Diffusion Coefficient vs GP;

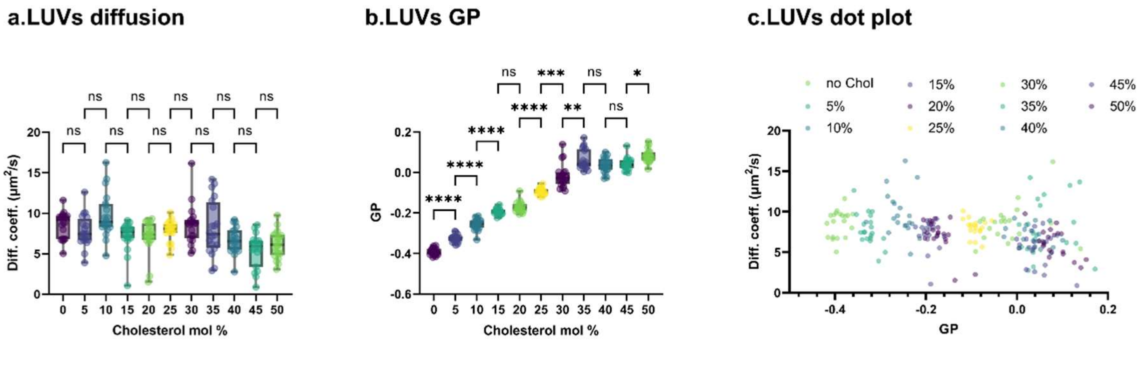

Supplementary Figure 14. LUVs with different content of cholesterol. LUVs that consist of POPC with different mol % of cholesterol were profiled. **a.** Diffusion coefficients of such

LUVs; **b.** GP of such LUVs; n=3 biological replicate, n=20 technical replicates, box = 25-75%, whiskers = min-max, line marks mean value), statistical significance was probed by Kruskal-Wallis nonparametric test with multiple comparisons, **** corresponds to p<0.0001, *** corresponds to p<0.001, ** corresponds to p<0.01, * corresponds to p<0.05 ns to p>0.05 **c.** Dot plot of Diffusion Coefficient vs GP.

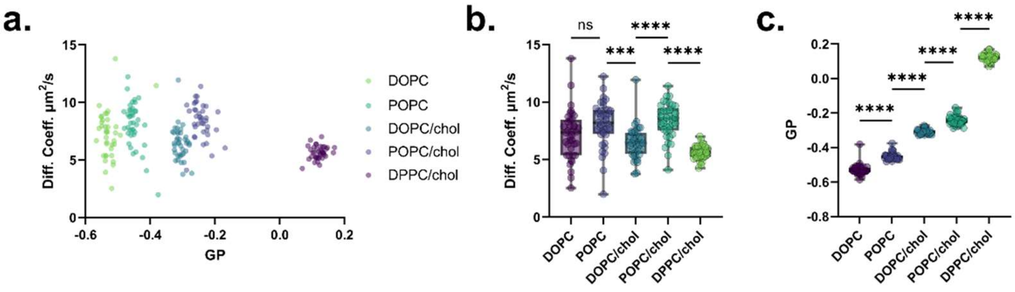

Supplementary Figure 15. Profiling of LUVs. LUVs were labeled by NR12S; **a.** Dot plot of Diffusion Coefficient vs GP; **b.** Diffusion coefficients of LUVs; n=3 biological replicate, n=40 technical replicates, box = 25-75%, whiskers = min-max, line marks mean value), statistical significance was probed by Kruskal-Wallis nonparametric test with multiple comparisons, **** corresponds to p<0.0001, *** corresponds to p<0.001, ** corresponds to p<0.01, * corresponds to p<0.05 ns to p>0.05 **c.** GP of LUVs.

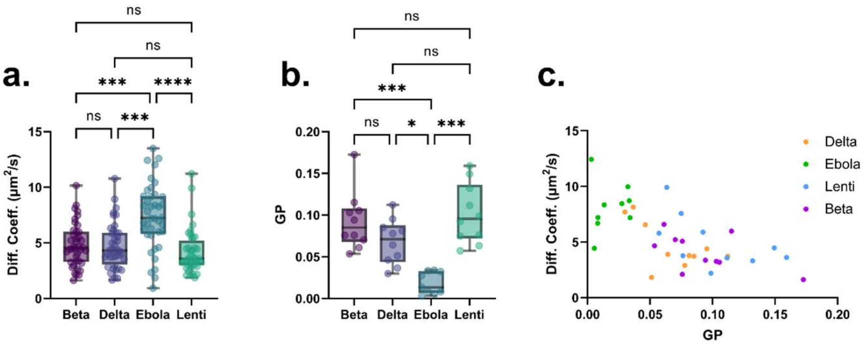

Supplementary Figure 16. Virus-like particles (VLPs). VLPs based on lentiviruses co transfected with SARS-COV-2 Spike glycoproteins (beta and delta mutations), ebola glycoprotein and lentiviruses without glycoproteins; **a.** VLPs diffusion; **b.** GP of VLPs; n=3 biological replicate, n=40 technical replicates in a, n=10 technical replicates in b box = 25-75%, whiskers = min-max, line marks mean value), statistical significance was probed by Kruskal-Wallis nonparametric test with multiple comparisons, **** corresponds to p<0.0001, *** corresponds to p<0.001, ** corresponds to p<0.01, * corresponds to p<0.05 ns to p>0.05 **c.** Dot plot of Diffusion Coefficient vs GP for VLPs;

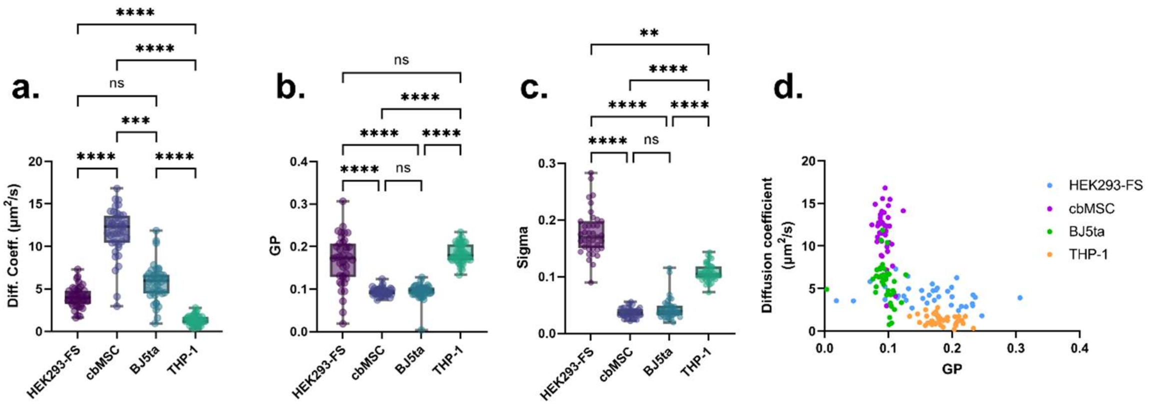

Supplementary Figure 17. Exosomes isolated from different cells. a. Exosome diffusion; **b.** GP of exosomes; **c.** Sigma of EVs; n=3 biological replicate, n=40 technical replicates, box = 25-75%, whiskers = min-max, line marks mean value), statistical significance was probed by Kruskal-Wallis nonparametric test with multiple comparisons, **** corresponds to p<0.0001, *** corresponds to p<0.001, ** corresponds to p<0.01, * corresponds to p<0.05 ns to p>0.05 **d.** Dot plot of Diffusion Coefficient vs GP for exosomes.

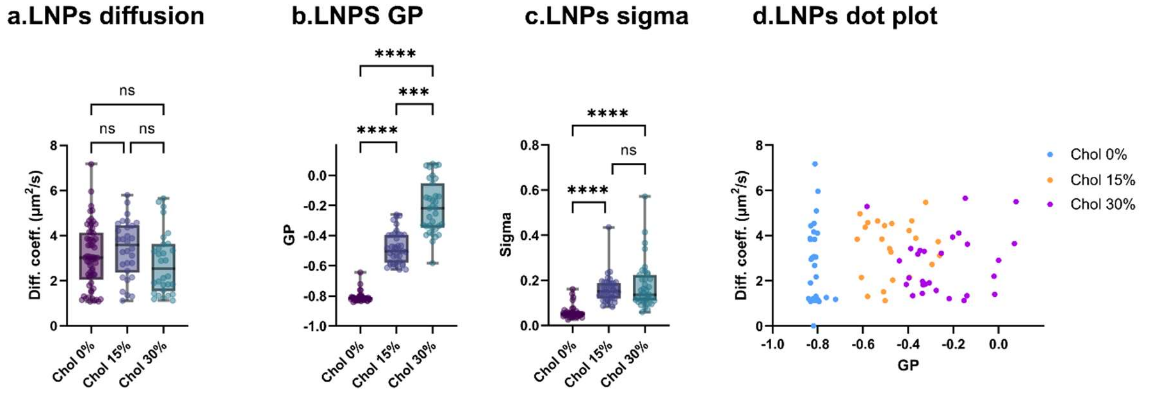

Supplementary Figure 18. LNPs with different content of cholesterol. a. Diffusion of lipid nanoparticles (LNPs); **b.** GP of LNPs; **c.** Sigma of LNPs; n=3 biological replicate, n=40 technical replicates, box = 25-75%, whiskers = min-max, line marks mean value), statistical significance was probed by Kruskal-Wallis nonparametric test with multiple comparisons, **** corresponds to p<0.0001, *** corresponds to p<0.001, ** corresponds to p<0.01, * corresponds to p<0.05 ns to p>0.05 **d.**Dot plot of LNPs;

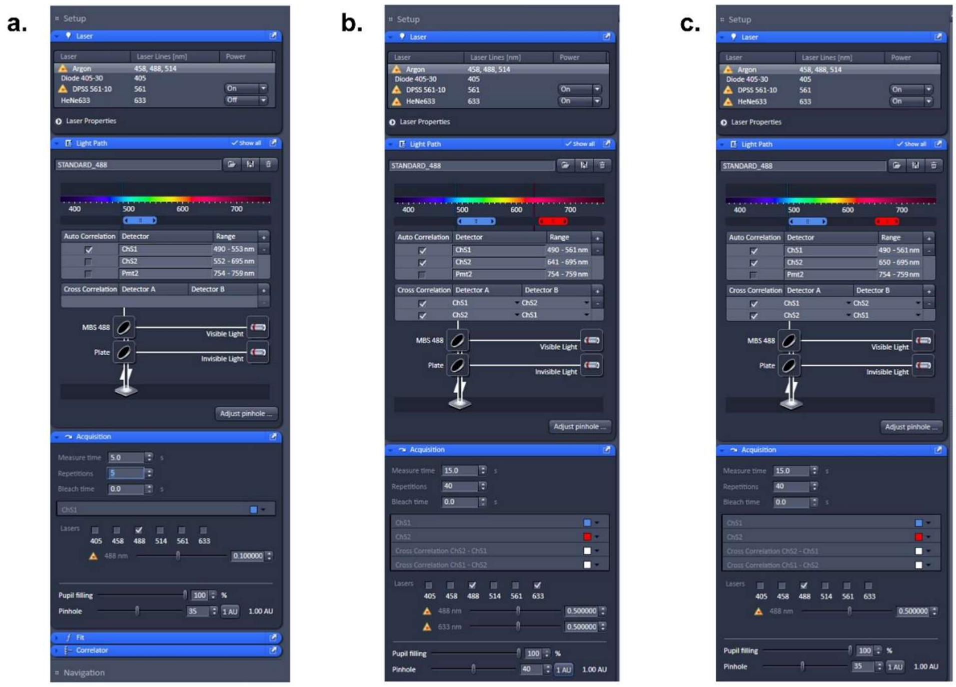

Supplementary Figure 19. Settings for Fluorescence Correlation Spectroscopy measurements for SPP. a. Settings for calibration with Alexa 488 in water. Excitation laser: 488 nm, detection window: 490-553 nm, 5s measurement time with 5 repetitions, laser power at 0.1%; **b.** Settings for two-color profiling with TF-chol and ASR-PE. Excitation lasers: 488 nm for TF-Chol and 633 nm for ASR-PE, detection windows: 490-561 nm for TF-Chol and 641-695 nm for ASR PE, 15s measurement time with 40 repetitions, laser powers at 0.5% **c.** Settings for biophysical profiling with ratiometric lipophilic probe NR12S. Excitation lasers: 488 nm, detection windows: 490-561 nm for green part of NR12S spectrum and 650-695 nm for red part of NR12S spectrum, 15s measurement time with 40 repetitions, laser power at 0.5%.
