# Supplementary material for: High-throughput measurement of the content and properties of nano-sized bioparticles with single-particle profiler
Source: Nat Biotechnol. 2023 Jun 12;42(4):587–90. doi: 10.1038/s41587-023-01825-5 (PMC11021190; doi:10.1038/s41587-023-01825-5)
Supplement: Supplementary file 2 — Reporting Summary [file 41587_2023_1825_MOESM2_ESM.pdf]

nature research

Corresponding author(s): Erdinc Sezgin

Last updated by author(s): 08/05/2023

Reporting Summary

Nature Research wishes to improve the reproducibility of the work that we publish. This form provides structure for consistency and transparency in reporting. For further information on Nature Research policies, see our Editorial Policies and the Editorial Policy Checklist.

Statistics

For all statistical analyses, confirm that the following items are present in the figure legend, table legend, main text, or Methods section.

n/a	Confirmed	
☐	☒	The exact sample size (n) for each experimental group/condition, given as a discrete number and unit of measurement
☐	☒	A statement on whether measurements were taken from distinct samples or whether the same sample was measured repeatedly
☐	☒	The statistical test(s) used AND whether they are one- or two-sided<br>*Only common tests should be described solely by name; describe more complex techniques in the Methods section.*
☒	☐	A description of all covariates tested
☒	☐	A description of any assumptions or corrections, such as tests of normality and adjustment for multiple comparisons
☐	☒	A full description of the statistical parameters including central tendency (e.g. means) or other basic estimates (e.g. regression coefficient) AND variation (e.g. standard deviation) or associated estimates of uncertainty (e.g. confidence intervals)
☐	☒	For null hypothesis testing, the test statistic (e.g. F, t, r) with confidence intervals, effect sizes, degrees of freedom and P value noted<br>*Give P values as exact values whenever suitable.*
☒	☐	For Bayesian analysis, information on the choice of priors and Markov chain Monte Carlo settings
☒	☐	For hierarchical and complex designs, identification of the appropriate level for tests and full reporting of outcomes
☒	☐	Estimates of effect sizes (e.g. Cohen's d, Pearson's r), indicating how they were calculated

Our web collection on statistics for biologists contains articles on many of the points above.

Software and code

Policy information about availability of computer code

Data collection	For data acquistion, microscopy software ZEN 2012 SP5 FP3 (black), version 14.0.21.0201 was used.
Data analysis	For data analysis, the home-made python-based program was developed. The following freely available packages were used: 'tkinter','matplotlib','lmfit', 'ttkwidgets', 'scipy', 'seaborn'. For plotting and statistical analysis GraphPad Prism 9 (version 9.4.0) was used

For manuscripts utilizing custom algorithms or software that are central to the research but not yet described in published literature, software must be made available to editors and reviewers. We strongly encourage code deposition in a community repository (e.g. GitHub). See the Nature Research guidelines for submitting code & software for further information.

Data

Policy information about availability of data

All manuscripts must include a data availability statement. This statement should provide the following information, where applicable:

- Accession codes, unique identifiers, or web links for publicly available datasets
- A list of figures that have associated raw data
- A description of any restrictions on data availability

Raw data available at FigShare 10.17044/scilifelab.20338869

Field-specific reporting

Please select the one below that is the best fit for your research. If you are not sure, read the appropriate sections before making your selection.

☒ Life sciences ☐ Behavioural & social sciences ☐ Ecological, evolutionary & environmental sciences

For a reference copy of the document with all sections, see nature.com/documents/nr-reporting-summary-flat.pdf

Life sciences study design

All studies must disclose on these points even when the disclosure is negative.

Sample size	All the data shown in the paper have been replicated at least 3 times. Sample sizes are predetermined by the comparative nature of our questions (e.g., nanobody A vs nanobody B, hence two sample groups).
Data exclusions	GP values of exactly 1 or exacly -1 were excluded, sigma values of 0 were excluded, diffusion coefficients calculated from FCS curves that showed prescens of sample aggregates were excluded.
Replication	Biological samples from different donors were treated as separate datasets. Not less then 10 and not more then 60 technical replicates were performed depending on the number of particles retrieved from the biological material. Number of technical replicates for every dataset is stated in the figure legends.
Randomization	Our comparative data (no directed null hypothesis) do not require randomization.
Blinding	Our comparative data (no directed null hypothesis) do not require blinding.

Reporting for specific materials, systems and methods

We require information from authors about some types of materials, experimental systems and methods used in many studies. Here, indicate whether each material, system or method listed is relevant to your study. If you are not sure if a list item applies to your research, read the appropriate section before selecting a response.

Materials & experimental systems

n/a	Involved in the study	
☒	☐	Antibodies
☐	☒	Eukaryotic cell lines
☒	☐	Palaeontology and archaeology
☒	☐	Animals and other organisms
☒	☐	Human research participants
☒	☐	Clinical data
☒	☐	Dual use research of concern

Methods

n/a	Involved in the study	
☒	☐	ChIP-seq
☒	☐	Flow cytometry
☒	☐	MRI-based neuroimaging

Eukaryotic cell lines

Policy information about cell lines

Cell line source(s)	Human Embryonic Kidney FS (HEK293-FS, Thermofisher R79007); Acute monocytic leukemia Human (THP-1, ATCC TIB-202); Human Skin Fibroblast (BJ-5ta, ATCC CRL-4001); HEK293T (ATCC CRL-3216); Cord Blood Mesenchymal Stromal Cells (cbMSC; ATCC PCS-500-010)
Authentication	Authentication was done by the supplier.
Mycoplasma contamination	All cells were tested negative for mycoplasma.
Commonly misidentified lines (See ICLAC register)	N/A
